# COMPLEMENTARY FEEDING INDICATORS FOR CHILDREN AGED 6 TO 23 MONTHS ACCORDING TO BREASTFEEDING STATUS

**DOI:** 10.1590/1984-0462/2021/39/2019408

**Published:** 2020-10-21

**Authors:** Daniella Garcia Vidal Rodrigues Leonez, Angélica Rocha de Freitas Melhem, Daniele Gonçalves Vieira, Débora Falleiros de Mello, Paula Chuproski Saldan

**Affiliations:** a Universidade Estadual do Centro-Oeste, Guarapuava, PR, Brazil.; b School of Nursing of Ribeirão Preto, Universidade de São Paulo, Ribeirão Preto, SP, Brazil.

**Keywords:** Indicators, Infant, Breast feeding, Indicadores, Lactente, Aleitamento materno

## Abstract

**Objective:**

To verify if there are differences among the complementary feeding indicators of children aged 6-23 months according to the breastfeeding status.

**Methods:**

A cross-sectional study was carried out with 1,355 children aged 6-23 months in 2012 to evaluate five indicators proposed by the World Health Organization (WHO) and modified in accordance with Brazilian’s recommendations “Ten steps to a healthy feeding: a feeding guide for children under two years old”. The indicators used were: I. Introduction of solid, semi-solid or soft foods; II. Minimum dietary diversity; III. Minimum meal frequency; IV. Minimum acceptable diet, and V. Consumption of iron-rich foods. To verify differences between the complementary feeding indicators according to breastfeeding status, the F-statistic was used, with p≤0.05 meaning significant.

**Results:**

Indicators I, II, and V were similar among breastfed and non-breastfed children; however, indicators III and IV presented a higher proportion of adequacy for non-breastfed children, with 94.9% (CI95% 93.2-96.2) *versus* 40.3% (CI95% 33.2-47.9) for indicator III, and 57.3% (CI95% 53.2-61.2) *versus* 23.1% (CI95% 17.4-30.1) for indicator IV.

**Conclusions:**

Non-breastfed children have better complementary feeding status, but the indicator III takes into account non-breast milk as a meal for non-breastfed children, which increased the number of dairy meals and influenced indicator IV (calculated from indicators II and III).

## RESUMO

**Objetivo:**

Verificar se existem diferenças entre os indicadores de alimentação complementar de crianças de 6 a 23 meses segundo o estado de amamentação.

**Métodos:**

Estudo transversal realizado em 2012 com 1.355 crianças de 6 a 23 meses de idade com avaliação de cinco indicadores propostos pela Organização Mundial da Saúde (OMS) e modificados com base nas recomendações dos *Dez passos para uma alimentação saudável*: *guia alimentar para crianças menores de dois anos*, do Ministério da Saúde. Os indicadores utilizados foram: I. Introdução de alimentos sólidos, semissólidos ou pastosos; II. Diversidade mínima da dieta; III. Frequência mínima das refeições; IV. Dieta mínima aceitável; e V. Consumo de alimentos ricos em ferro. Para verificar diferenças entre os indicadores de alimentação complementar segundo o estado de amamentação foi empregada a estatística F, sendo significante p≤0,05.

**Resultados:**

Os indicadores I, II e V foram semelhantes entre as crianças amamentadas e não amamentadas, porém os indicadores III e IV apresentaram maior proporção de adequação para as não amamentadas: para o indicador III, 94,9% (intervalo de confiança de 95% - IC95% 93,2-96,2) *versus* 40,3% (IC95% 33,2-47,9), e para o indicador IV, 57,3% (IC95% 53,2-61,2) *versus* 23,1% (IC95% 17,4-30,1).

**Conclusões:**

As crianças não amamentadas apresentaram melhor situação de alimentação complementar, porém o indicador III leva em consideração o leite não materno como refeição para crianças não amamentadas, o que elevou o número de refeições lácteas e influenciou o indicador IV, calculado a partir dos indicadores II e III.

## INTRODUCTION

Feeding is essential in the first years of life to the formation of eating habits, with short and long-term implications for the child’s health.^1^ Thus, the recommendation is for infants to be exclusively breastfed in the first six months of their lives and, as from that age, receive adequate and healthy complementary foods, with maintenance of breastfeeding for two years or more.^1^ ^,^ ^2^ ^,^ ^3^

The feeding of the nursing mother also influences the child’s habits, because flavors and aromas of food are passed on to the infant via breast milk (BM). Exposure to a diversity of foods in the complementary feeding (CF) phase can influence the dietary preferences of the subsequent phases.^2^ ^,^ ^3^ A longitudinal study, which evaluated the variety of fruits and vegetables consumed by school-aged children, found that the varied consumption of vegetables was influenced by maternal preferences; the variety of fruits was influenced by the longer duration of breastfeeding (BF) and the variety or exposure of the fruit consumed in the first two years of life.^4^

In order to assess CF, the World Health Organization (WHO) recommends using five indicators, for enabling international comparisons.^5^ ^,^ ^6^ The Brazilian Ministry of Health (MH) prepared a document to evaluate CF, which follows the guidelines by the WHO and also contains five indicators, adapted to the Brazilian reality, modified based on the “Ten steps to a healthy feeding: a feeding guide for children under two years old”.^7^

Despite the publication of CF indicators, few studies in the national literature using this proposal are identified. International studies that used the WHO indicators proved to be useful to assess the feeding of children in undeveloped countries with high rates of malnutrition, such as African and Asian countries.^8^ ^,^ ^9^ ^,^ ^10^ ^,^ ^11^ ^,^ ^12^ ^,^ ^13^ ^,^ ^14^ ^,^ ^15^ ^,^ ^16^ ^,^ ^17^ ^,^ ^18^ A cross-sectional study carried out in Paraná State evaluated the CF of children aged 6 to 23 months using the WHO indicators, and showed that they were not sensitive to detect feeding problems at this stage of life, with their adequacy exceeding 85%. However, the same study, when evaluating CF based on indicators developed according to national recommendations on CF showed a less favorable situation.^19^

Thus, it seems to be important to evaluate CF by following national recommendations for children,^19^ in addition to assessing whether the indicators of CF for children who are being breastfed differ or not from the indicators of non-breastfed children, given the superiority of BM in relation to non-breastfed milk.^20^ Thus, the objective of the present study was to verify whether there are differences between the CF indicators elaborated from the national recommendations for children aged 6 to 23 months according to the breastfeeding status.

## METHOD

Cross-sectional study carried out during the National Vaccination Campaign against Poliomyelitis in Guarapuava City, Paraná State, 2012. The study population was the group of children under 2 years old who attended the vaccination posts in the urban and rural areas of the municipality. With information on the vaccinated population in the 2011 campaign, grouped into two age groups (<1 year old and 1-5 years old), the sample was stratified into age groups: children under 1 year old and children aged 12 to 23 months. The number of children between 12 and 23 months old was estimated based on the population of children between 1 and 5 years old, assuming homogeneous distribution among the age groups (1, 2, 3, and 4 years old).

The sample size was estimated to analyze BF, and CF indicators in different age groups. For children under 1 year old, the prevalence of exclusive breastfeeding in children under 6 months old was used, with a parameter of 40% between 2 and 3 months, according to a local study, and a sample error of 9%.^21^ For children aged 12 to 23 months, the indicator of continuity of breastfeeding for children aged 1 year (40%) was adopted, considering the age range of 12 to 15 months and sampling error of 6%. Sample size estimates were obtained by applying the algebraic expression by Lwanga and Lemeshow and, subsequently, a non-response adjustment of 5% and a design effect of 1.4 to compensate for the loss of precision of the sample by clusters.^22^ The final sample calculation for children under 1 year old and from 12 to 23 months old was 1,005 and 1,129, respectively.

The study adopted cluster sampling, with a two-stage draw.^23^ In the first stage, vaccination stations were drawn according to the number of children vaccinated in 2011. In the second stage, children were randomly selected in the vaccination queue of each station. In the total, 32 vaccination stations were drawn, and for each station, the fraction of the draw required to interview approximately 35 carers of children under 1 year old and 38 of children between 12 and 23 months old was estimated.

Data collection was carried out from 11 to 29 June 2012, by 118 trained university students. The data collection instrument was a questionnaire based on and modified from the one applied in the II Survey on the Prevalence of BF in the Brazilian Capitals and the Federal District in 2008.^22^ This questionnaire included 67 questions regarding the characteristics of the participants, the use of the health service and the children’s food consumption (BM, other types of milk, food groups, food consistency). The questions about food were based on all the probable foods the child ate the day before the interview (24-hour recall),^5^ asked to the children’s carers before vaccination.

CF was evaluated based on five indicators proposed by the WHO⁶, which were adapted according to the document by the MH entitled “Ten steps to a healthy feeding: a feeding guide for children under two years old”, namely:^19^ ^,^ ^24^

- Introduction of solid, semi-solid or soft foods: proportion of children aged 6 to 8 months who received these foods. For the calculation, the proportion of children who received fruits, salt food (vegetable puree, rice, beans, pasta, meat) and the consistency of the food (mashed or in pieces) was taken into account.
- Minimum dietary diversity: proportion of children aged 6 to 23 months who received food from six food groups: (i) grains, roots and tubers; (ii) legumes; (iii) BM, non-breast milk and dairy products; (iv) meat and eggs; (v) vegetables; and (vi) fruits.
- Minimum frequency of meals: proportion of children aged 6 to 23 months who received salt food in the appropriate consistency (mashed or in pieces), including dairy meals for non-breastfed children, and the minimum number of times (two times for children aged 6 to 8 months being breastfed; three times for children aged 9 to 23 months being breastfed; and four times for children aged 6 to 23 months not being breastfed).^5^
- Minimum acceptable diet: proportion of children aged 6 to 23 months who received a minimum acceptable diet. It is considered a composite indicator, calculated from indicators II and III.
- Consumption of iron-rich foods: proportion of children aged 6 to 23 months who consumed meat and beans.

The indicators were calculated according to the children’s breastfeeding status: breastfed, mixed feeding and non-breastfed. Children considered to be breastfed received only BM; those in mixed feeding, BM and another milk (pasteurized milk, long-life milk, fresh cow milk, regular powdered milk, infant formula, milk offered by the government - pasteurized and enriched with vitamins A and D, iron and zinc - and milk from other animals); and non-breastfed children, who only received another milk.^25^ In addition, the indicators were calculated for the proportion of children aged 6 to 23 months and disaggregated according to the children’s age group (6-11, 12-17, and 18-23 months).^6^

Responses like “do not know” or “not informed” by the respondents were treated as missing data and were not considered for the calculation of the indicators. All estimates were calculated considering the design effect (survey module) and sample weights, according to the age group domains when the estimates included children of both age groups (<1 year and 12-23 months). Data analysis was performed using the Stata program, version 12.0 (Stata Corp., College Station, Texas, United States). The estimates were presented in points and 95% confidence intervals (95%CI). The F-statistic (p≤0.05) was used to verify differences between the CF indicators of children according to the breastfeeding status and to the age groups analyzed.

The study was approved by the Research Ethics Committee (CEP) of the School of Nursing of Ribeirão Preto at Universidade de São Paulo - EERP-USP (Official Letter No. 253/2012, of June 11, 2012). All participants were informed about the study and those who agreed to participate gave their verbal consent.

## RESULTS

In this study, data were collected from 1,848 children. However, 18 (1%) were excluded because they did not live in Guarapuava City, and 16 (0.9%) due to inconsistency in age or lack of information about the date of birth. The number of refusals was 149 children (8.1%), and the response rate was 90.8%. For the calculation of CF indicators, those aged 6 to 23 months were considered, totaling 1,355 children. Children under 6 months old (n=459, 25.3% of the sample) were not considered in this study, given the recommendation to introduce CF as from 6 months and the calculation of CF indicators to consider the age group from 6 to 23 months and 29 days.

Of the 1,355 children who participated in the survey, 476 (31.1%) were between 6 and 11 months old; 490 (36.1%), between 12 and 17; and 389 (28.7%), between 18 and 23. Of these children, 706 (52.1%) were girls; 877 (64.7%) attended the public health system; 591 (43.6%) were daughters of mothers who had between 8 and 11 years of schooling; and most, 1,230 (90.8%), lived in the urban area. Regarding the children’s breastfeeding status, 223 (16.5%) were breastfed, 300 (22.1%) were on mixed feeding, 793 (58.5%) were not breastfed, and 39 (2.9%) did not have such information to report.

According to Table 1, the indicators “I. Introduction of solid, semi-solid or soft foods”; “II. Minimum dietary diversity”; and “V. Consumption of iron-rich foods” showed no difference in the proportions of adequacy according to the children’s breastfeeding status. The indicators “III. Minimum frequency of meals”; and “IV. Minimum acceptable diet” showed a higher proportion of adequacy among non-breastfed children (p<0.0001).

**Table 1:** Proportion of adequacy for complementary feeding indicators according to the breastfeeding status of children aged 6 to 23 months in Guarapuava City, Paraná State, 2012.

Complementary feeding indicators	Adequacy for breastfed children*	Adequacy for children with mixed feeding*	Adequacy for non-breastfed children*	p-value
I. Introduction of solid, semi-solid or soft foods**	65.1 (49.1-78.3)	65.2 (52.6-76.0)	65.1 (52.9-75.6)	0.999
II. Minimum dietary diversity	51.5 (43.3-59.7)	60.1 (53.2-66.6)	60.6 (57.1-64.1)	0.099
III. Minimum frequency of meals	40.3 (33.2-47.9)	33.2 (27.9-38.9)	94.9 (93.2-96.2)	<0.001
IV. Minimum acceptable diet	23.1 (17.4-30.1)	22.6 (17.6-28.6)	57.3 (53.2-61.2)	<0.001
V. Consumption of iron-rich foods	69.8 (62.5-76.3)	73.6 (67.2-79.2)	76.9 (73.9-79.6)	0.150

*Data expressed in % (95% confidence interval); **Considering the age group from 6 to 23 months, except for indicator I (for children from 6 to 8 months old).

When analyzing the indicators disaggregated by age group (6-11, 12-17 and 18-23 months) and breastfeeding status, the same differences were observed in the proportions of adequacy for indicators III and IV (p<0.0001), with a better situation for non-breastfed children (Tables 2, 3 and 4). For indicator IV in the 6 to 11-month age group, better proportions of adequacy were found among non-breastfed children, followed by mixed feeding and breastfed children, with a linear trend of p<0.001 (Table 2).

**Table 2:** Proportion of adequacy for complementary feeding indicators according to the breastfeeding status of children aged 6 to 11 months in Guarapuava City, Paraná State, 2012.

Complementary feeding indicators	Adequacy for breastfed children*	Adequacy for children with mixed feeding*	Adequacy for non-breastfed children*	p-value
II. Minimum dietary diversity	40.0 (30.5-50.3)	44.4 (34.5-54.9)	51.3 (42.7-59.8)	0.239
III. Minimum frequency of meals	49.6 (42.0-57.2)	47.8 (39.5-56.2)	88.4 (81.3-93.0)	<0.001
IV. Minimum acceptable diet	19.5 (13.6-27.1)	21.9 (15.2-30.5)	46.1 (37.0-55.5)	<0.001**
V. Consumption of iron-rich foods	50.0 (41.3-58.7)	49.6 (38.6-60.7)	59.7 (52.1-66.9)	0.164

*Data expressed in % (95% confidence interval); **Test for linear trend.

**Table 3:** Proportion of adequacy for complementary feeding indicators according to the breastfeeding status of children aged 12 to 17 months in Guarapuava City, Paraná State, 2012.

Complementary feeding indicators	Adequacy for breastfed children*	Adequacy for children with mixed feeding*	Adequacy for non-breastfed children*	p-value
II. Minimum dietary diversity	52.4 (40.0-64.5)	60.6 (51.6-68.9)	57.4 (52.0-62.6)	0.534
III. Minimum frequency of meals	32.8 (21.4-46.8)	27.1 (20.4-35.1)	93.5 (89.7-95.9)	<0.001
IV. Minimum acceptable diet	20.3 (11.7-32.9)	19.6 (14.3-26.4)	53.7 (48.4-59.0)	<0.001
V. Consumption of iron-rich foods	72.3 (62.9-80.1)	78.1 (68.7-85.3)	73.4 (68.5-77.7)	0.501

*Data expressed in % (95% confidence interval).

**Table 4:** Proportion of adequacy for complementary feeding indicators according to the breastfeeding status of children aged 18 to 23 months in Guarapuava City, Paraná State, 2012.

Complementary feeding indicators	Adequacy for breastfed children*	Adequacy for children with mixed feeding*	Adequacy for non-breastfed children*	p-value
II. Minimum dietary diversity	60.0 (39.5-77.5)	70.0 (56.9-80.5)	66.0 (59.1-72.3)	0.646
III. Minimum frequency of meals	45.7 (30.4-61.9)	35.3 (24.4-47.9)	97.8 (95.5-98.9)	<0.001
IV. Minimum acceptable diet	31.4 (18.4-48.3)	29.4 (19.0-42.5)	63.4 (56.0-70.1)	<0.001
V. Consumption of iron-rich foods	82.9 (66.0-92.3)	82.0 (65.9-91.5)	84.1 (80.4-87.2)	0.928

*Data expressed in % (95% confidence interval).

## DISCUSSION

This study made it possible to evaluate CF indicators according to the breastfeeding status of children from 6 to 23 months old. Food consumption was assessed in the 24 hours prior to the survey, thus preventing memory bias and making it possible to outline a profile of children’s food.^5^

Regarding the external validity of research, it can be assessed with the high coverage of the Vaccination Campaign against Poliomyelitis in Guarapuava City, from 2012, which reached 99% of the children in the municipality, and with the similar profile of the sample studied with data from the Information System of Live Births (*Sistema de Informação de Nascidos Vivos* - SINASC), 2012, for the municipality.^26^ ^,^ ^27^ Of the children studied, 88.3% were born with the adequate weight (≥2,500 g) and 51.3% through vaginal delivery (*versus* 91.2 and 46.7% of the reference population, respectively).^27^

In Brazil, few are the studies using these indicators comparing breastfed and non-breastfed children. However, a study that evaluated the food consumption patterns of 1,455 children aged 6 to 24 months, according to their breastfeeding status, showed that children who did not consume non-human milk were more likely to be on a healthy and diversified diet, having a consumption of foods rich in sugar, fat and salt less than children who consumed other types of milk.^28^ This result differs from that found in the present study in the item “minimum dietary diversity”, with no significant difference according to the breastfeeding status of children. However, low values (ranging from 51.5 to 60.6%) were observed for all breastfeeding states.

A study carried out in São Paulo^29^ with 14,327 children aged 6 to 12 months analyzed the indicators “minimum dietary diversity” and “minimum acceptable diet”, and found that most infants had a negative rating for these indicators (68.2 and 71.1%, respectively). Despite not comparing to the breastfeeding status and covering only the age group from 6 to 12 months, it is possible to observe unsatisfactory prevalence of the indicators, suggesting that most infants do not receive complementary foods in an adequate amount and consistency for their appropriate development, which is also observed in this study. This may occur because this age group is in the initial phase of CF, when children are still becoming familiar with food - in the other age groups evaluated in this study (12-17 and 18-23 months), better values for CF indicators can be verified.

In relation to the minimum dietary diversity, a study carried out in Barra Mansa City, Rio de Janeiro State, with 580 children aged 6 to 12 months, found that 35.5% of them received diversified food, which was less frequent (22.9 %) in the age group from 6 to 7 months, reaching 39.3% of children aged 8-9 months, and 42.3% of those aged 10-11 months, also showing improvement in this indicator as the child’s age range increases.^30^

In the international scenario, a study on CF practices in 85 countries showed variations in indicators at the global and regional levels, with the minimum frequency of meals, the minimum dietary diversity, and the minimum acceptable global diet for all children being 52, 29 and 16%, respectively. The best results obtained were for Latin America and the Caribbean, with 78 and 73%, respectively, with no data on minimum acceptable diet for that region.^13^

A study carried out in Tanzania, Africa, which evaluated the CF indicators of 2,402 children aged 6 to 23 months, comparing their breastfeeding status, demonstrated that the introduction of solid, semi-solid and soft foods was suitable for 92.3% of children between 6 and 8 months in all breastfeeding states. In terms of “minimum dietary diversity” and “minimum frequency of meals”, the indicators were better for non-breastfed children (45.3; 34.2%) than for breastfed children (36.9; 11.4%), showing a result similar to that found in the present study, but with unsatisfactory values.^8^

A study conducted in Pakistan, South Asia, with 2,827 children aged 6 to 23 months reported that the introduction of solid, semi-solid and soft foods did not differ between breastfed (66%) and non-breastfed (71%) children. Nonetheless, the minimum frequency of meals was significantly lower among breastfed children when compared to non-breastfed children, similar to that found in the present study.^14^ Another study carried out in Afghanistan, central Asia, with only breastfed children, aged 6 to 23 months, found that more than half reached the indicators “food introduction” and “minimum frequency of meals”, whereas the indicators “minimum dietary diversity” and “minimum acceptable diet” showed lower percentages of adequacy (23 and 18%, respectively), differing of the present study, which reported lower adjustments for the frequency of meals (40.3%), followed by the minimum acceptable diet (23.1%).^18^

A study conducted in Ghana, Africa, with 822 children aged 6 to 23 months found that 75% of children aged 6 to 8 months had an adequate introduction of solid, semi-solid or soft foods; just over half reached the minimum criterion for food diversity; less than half met the criterion for minimum frequency of meals; and less than a third had access to the minimum acceptable diet, proving a high rate of inadequacy of CF indicators in this country.^9^ As much as this study did not compare breastfed and non-breastfed children, it can be observed that the values found by the researchers are close to those found in research with breastfed children - it should be noted, however, that the indicators from the Ghana study were based on the WHO criteria, and those of the present study, on the WHO indicators modified from Brazilian dietary recommendations for children under two years old. Thus, the similarity that stands out among the studies must be carefully analyzed, because the indicators were not elaborated in the same way.

A limitation of the present study was the fact that indicator III considers non-breast milk as a meal for non-breastfed children, which increased the number of dairy meals and, consequently, influenced indicator IV, which is calculated from indicators II and III. Hence, non-breastfed children who consumed several dairy meals throughout the day may have had more adequacy of indicators III and IV, but not necessarily from meals such as lunch or dinner. Conversely, the number of times the child received BM is not counted for the calculation of the minimum frequency of meals indicator, because, according to the WHO, the CF indicators are used to reflect foods other than BM, and non-breastfed children may show results of some CF indicators better than breastfed children.^6^ Another limitation of the present study was the use of the 24-hour recall, which may not reflect the children’s usual diet, but this is still the method recommended by the WHO to assess feeding practices of children under two years old.^5^ Moreover, the lack of evaluation of the maternal diet for breastfed children and the lack of family evaluation of the diet, which can also influence the food offered to the child, is highlighted.

This type of study is relevant to sensibly delineate the evaluated indicators and the need to invest in public policies and socio-educational measures to improve these indexes, considering that CF has a significant impact on the child’s life in the short, medium and long terms. Given the scarcity of research in Brazil evaluating CF indicators developed based on national recommendations and guidelines and according to the children’s breastfeeding status, there is a need for further studies to confirm the findings of research.

In the present study, non-breastfed children had better indicators of CF (“III. Minimum frequency of meals” and “IV. Minimum acceptable diet”) when compared to children breastfed only with BM or mixed feeding, for all age groups analyzed.

## References

[1] Brazil - Ministério da Saúde. Secretaria de Atenção à Saúde.
Departamento de Atenção Básica (2015). Saúde da criança: aleitamento materno e alimentação
complementar.

[2] World Health Organization. Pan American Health Organization (2003). Guiding principles for complementary feeding of the breastfed
child.

[3] Brazil - Ministério da Saúde. Secretaria de Atenção Primária à
Saúde. Departamento de Promoção da Saúde (2019). Guia alimentar para crianças brasileiras menores de 2 anos.

[4] Skinner JD, Carruth BR, Bounds W, Ziegler P, Reidy K (2002). Do food-related experiences in the first 2 years of life predict
dietary variety in school-age children?. J Nutr Educ Behav.

[5] World Health Organization (2008). Indicators for assessing infant and young child feeding practices:
conclusions of a consensus meeting held 6-8 November 2007.

[6] World Health Organization (2010). Indicators for assessing infant and young child feeding practices: part
2 measurement.

[7] Brazil - Ministério da Saúde. Secretaria de Atenção à Saúde.
Departamento de Atenção Básica (2015). Orientações para avaliação de marcadores de consumo alimentar na atenção
básica.

[8] Victor R, Surinder KB, Agho KE, Dibley MJ (2014). Factors associated with inappropriate complementary feeding
practices among children aged 6-23 months in Tanzania. Matern Child Nutr.

[9] Issaka AI, Agho KE, Burns P, Page A, Dibley MJ (2015). Determinants of inadequate complementary feeding practices among
children aged 6-23 months in Ghana. Public Health Nutr.

[10] Ogbo FA, Page A, Idoko J, Claudio F, Agho KE (2015). Trends in complementary feeding indicators in Nigeria,
2003-2013. BMJ Open.

[11] Issaka AI, Agho KE, Page AN, Burns PL, Stevens GJ, Dibley MJ (2015). Comparisons of complementary feeding indicators among children
aged 6-23 months in Anglophone and Francophone West African
countries. Matern Child Nutr.

[12] Khor GL, Tan SY, Tan KL, Chan PS, Amarra MS (2016). Compliance with WHO IYCF indicators and dietary intake adequacy
in a sample of Malaysian infants aged 6-23 months. Nutrients.

[13] White JM, Bégin F, Kumapley R, Murray C, Krasevec J (2017). Complementary feeding practices: current global and regional
estimates. Matern Child Nutr.

[14] Na M, Aguayo VM, Arimond M, Stewart CP (2017). Risk factors of poor complementary feeding practices in Pakistani
children aged 6-23 months: a multilevel analysis of the Demographic and
Health Survey 2012-2013. Matern Child Nutr.

[15] Na M, Aguayo VM, Arimond M, Dahal P, Lamichhane B, Pokharel R (2018). Trends and predictors of appropriate complementary feeding
practices in Nepal: an analysis of national household survey data collected
between 2001 and 2014. Matern Child Nutr.

[16] Duan Y, Yang Z, Lai J, Yu D, Chang S, Pang X (2018). Exclusive breastfeeding rate and complementary feeding indicators
in China: a national representative survey in 2013. Nutrients.

[17] Nguyen PH, Avula R, Headey D, Tran LM, Ruel MT, Menon P (2018). Progress and inequalities in infant and young child feeding
practices in India between 2006 and 2016. Matern Child Nutr.

[18] Na M, Aguayo VM, Arimond M, Mustaphi P, Stewart CP (2018). Predictors of complementary feeding practices in Afghanistan:
analysis of the 2015 Demographic and Health Survey. Matern Child Nutr.

[19] Saldan PC, Venancio SI, Saldiva SR, Mello DF (2016). Proposal of indicators to evaluate complementary feeding based on
World Health Organization indicators. Nurs Health Sci.

[20] Victora CG, Bahl R, Barros AJ, França GV, Horton S, Krasevec J (2016). Breastfeeding in the 21st century: epidemiology, mechanisms, and
lifelong effect. Lancet.

[21] Brecailo MK, Corso AC, Almeida CC, Schmitz BA (2010). Factors associated with exclusive breastfeeding in Guarapuava,
Paraná, Brazil. Rev Nutr.

[22] Brazil - Ministério da Saúde. Secretaria de Atenção à Saúde.
Departamento de Ações Programáticas e Estratégicas (2009). II Pesquisa de prevalência de aleitamento materno nas capitais
brasileiras e Distrito Federal.

[23] Silva NN (2004). Amostragem probabilística: um curso introdutório.

[24] Brazil - Ministério da Saúde. Secretaria de Atenção à Saúde.
Departamento de Atenção Básica (2010). Dez passos para uma alimentação saudável: guia alimentar para menores de
dois anos: um guia para o profissional da saúde na atenção básica.

[25] Saldan PC, Venancio SI, Saldiva SR, Vieira DG, Mello DF (2017). Milk consumption in infants under one year of age and variables
associated with non‑maternal milk consumption. Rev Paul Pediatr.

[26] Brazil - Ministério da Saúde. Sistema de Informação do Programa
Nacional de Imunizações Campanha nacional de vacinação contra poliomielite 2012.

[27] Brazil - Ministério da Saúde. Departamento de Informática do Sistema
Único de Saúde (DATASUS) Nascidos vivos - Paraná.

[28] Bortolini GA, Giugliani ER, Gubert MB, Santos LM (2019). Breastfeeding is associated with children’s dietary diversity in
Brazil. Ciênc Saúde Coletiva.

[29] Passanha A, Benício MH, Venâncio SI (2020). Characterization of the food consumption of infants in the State
of São Paulo between six to twelve months of age. Ciênc Saúde Coletiva.

[30] Oliveira MI, Rigotti RR, Boccolini CS (2017). Factors associated with lack of dietary diversity in the second
semester of life. Cad Saude Colet.

